# Information Dynamics in Living Systems: Prokaryotes, Eukaryotes, and Cancer

**DOI:** 10.1371/journal.pone.0022085

**Published:** 2011-07-19

**Authors:** B. Roy Frieden, Robert A. Gatenby

**Affiliations:** 1 College of Optical Science, University of Arizona, Tucson, Arizona, United States of America; 2 Departments of Radiology and Integrated Mathematical Oncology, Moffitt Cancer Center, Tampa, Florida, United States of America; Governmental Technical Research Centre of Finland, Finland

## Abstract

**Background:**

Living systems use information and energy to maintain stable entropy while far from thermodynamic equilibrium. The underlying first principles have not been established.

**Findings:**

We propose that stable entropy in living systems, in the absence of thermodynamic equilibrium, requires an information extremum (maximum or minimum), which is invariant to first order perturbations. Proliferation and death represent key feedback mechanisms that promote stability even in a non-equilibrium state. A system moves to low or high information depending on its energy status, as the benefit of information in maintaining and increasing order is balanced against its energy cost. Prokaryotes, which lack specialized energy-producing organelles (mitochondria), are energy-limited and constrained to an information minimum. Acquisition of mitochondria is viewed as a critical evolutionary step that, by allowing eukaryotes to achieve a sufficiently high energy state, permitted a phase transition to an information maximum. This state, in contrast to the prokaryote minima, allowed evolution of complex, multicellular organisms. A special case is a malignant cell, which is modeled as a phase transition from a maximum to minimum information state. The minimum leads to a predicted power-law governing the *in situ* growth that is confirmed by studies measuring growth of small breast cancers.

**Conclusions:**

We find living systems achieve a stable entropic state by maintaining an extreme level of information. The evolutionary divergence of prokaryotes and eukaryotes resulted from acquisition of specialized energy organelles that allowed transition from information minima to maxima, respectively. Carcinogenesis represents a reverse transition: of an information maximum to minimum. The progressive information loss is evident in accumulating mutations, disordered morphology, and functional decline characteristics of human cancers. The findings suggest energy restriction is a critical first step that triggers the genetic mutations that drive somatic evolution of the malignant phenotype.

## Introduction

All living systems (i) have a local domain delimited by, for example, a cell membrane and (ii) maintain a stable, low level of entropy or disorder. The 2^nd^ law of thermodynamics requires entropy to increase with time but this is of a specifically global nature. Hence, property (i) of locality permits the system to have the beneficial property (ii) of low entropy. In compensation, the 2^nd^ law requires the locally low level of entropy be accompanied by export of an even larger amount of entropy into the system's exterior environment. In fact, this property is not unique to living systems – crystals, stars, and planets likewise represent locally ordered structures. However, living systems differ from other ordered structures in nature in that they

1. have regular internal entropy gradients with highly ordered structures like the cell membrane and chromosomes, interspersed with much less ordered components such as the cytoplasm;
2. maintain stable, local entropy using external energy far from thermodynamic equilibrium;
3. are capable of self replication;
4. store and use information in the form of genetic codes and, possibly, other ordered intracellular structures.
5. die

Thus, in brief living systems are low (but not minimum) entropy states that remain stable despite being far from thermodynamics equilibrium. This stability requires information to maintain internal mechanisms that efficiently convert energy to order. Proliferation and death provide positive and negative feedback that allow the system to maintain stability even though far from thermodynamic equilibrium.

We have previously demonstrated [1] that information in a biological context can be viewed as the capacity to facilitate work. Specifically, it directs and catalyzes the conversion of energy and substrate from the environment into specific macromolecules that, in turn, maintain the orderly structure of the cell. For example, information in DNA specifies the structure of proteins. Some proteins may function as enzymes in energy metabolism or lipid synthesis. Other macromolecules spontaneously self-assemble into higher order, low-energy, structural components of the cell such as proteins forming ribosome or lipids forming membranes.

This central role of information in maintaining a living system is unique in nature and may itself represent the most succinct possible definition of life. A quantitative metric of system ‘order’ or ‘complexity’ is ‘Fisher information’ [1]–[9] (they are all proportional, as below). The concept has been applied extensively to living and nonliving systems. Here our aim is to examine information dynamics that, *in the absence of thermodynamic equilibrium*, permit the formation and persistence of local pockets in which disorder is significantly less than that of the surrounding environment. These local pockets are viewed as living systems.

## Methods

### Two Postulates of Living Systems

Local increases in order can occur in non-living systems such as crystals. However, these physical systems invariably move toward a stable thermodynamic equilibrium state of low entropy and energy. In contrast with crystals, living systems maintain a stable state of low entropy that is far from thermodynamic equilibrium. Key system parameters that permit stability of the living system are information and energy. The latter must flow into the cell and then be converted to order through interactions with the former. However, we propose that living systems fundamentally must balance the benefits of increased information and complexity with the cost of acquiring and maintaining that information. Thus, living systems achieve not the lowest possible entropy state but, rather, an entropy level that permits proliferation within constraints of available information and energy [see Eqs. (2a,b)].

The preceding suggests the use of Fisher information, which is a measure of entropy and order (see below) and an indicator of energy cost, can be used to express the first principles of the thermodynamics of living systems, as follows:

- **Living systems are non-equilibrium open, but locally delimited, thermodynamic systems that use information to convert environmental energy to order. Survival of a living structure requires a stable state of order despite continuous thermal and mechanical perturbations.** The rapid (in geological time) appearance of life on earth and its durability since first appearing in the fossil record indicates that living systems represent a highly favorable state that can develop spontaneously and remain stable and robust despite a wide range of perturbations over billions of years.
- **The stability of a living system requires its information content to be maintained at an extremum.** Since their first derivative is 0, extreme points in system information dynamics tend to be stable to first-order system perturbations. We propose the robustness of living systems is presumptive evidence that its information state is at either a minimum or maximum value [See, in particular, material following Eq. (1)]. This will be seen to require a balance between the availability and cost of the energy in the environmental substrate of the system. The extreme values manifest biologically as follows:
  - Prokaryotes. The energy availability of prokaryotes is limited by their environmental substrate and the absence of specialized energy producing organelles. Limited energy availability constrains system dynamics, so that the information which, we saw, must be maintained at an extremum, can only be maintained at its *minimum* value. This minimum level satisfies requirement **I**.
  - Eukaryotes. Eukaryotes utilize specialized organelles for energy production including chloroplasts and mitochondria. These release the energy limitations that had constrained prokaryotes to an information minimum. In this state, information is maintained at a *maximum*. That is, when energy is abundant, the benefit of increased information and order exceeds the added cost. This information phase changes is reflected in the increased size of the cell and number of genes when compared to prokaryotes, as well as subsequent evolution to complex, multicellular organisms [10].

Postulates **I** and **II** are the basis for our thesis that Fisher information provides a blueprint for the growth and development of life. Intuitive and motivational reasons behind the use of these extreme effects are given later. But first, what is Fisher information?

### Fisher Information

Consider a system with a characteristic parameter whose value is sought by analysis of its data. The data are used to form a mathematical estimate of the parameter. The information 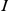 was originally defined by R.A. Fisher [11] as a measure of the quality of data about the parameter. Properties of Fisher information are developed in Supporting Material. Among these are its ‘local’ nature and shift-invariant form 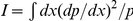, with 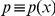 the probability density law defining the system with a coordinate 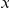. In general, the nature of 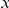 depends upon the application, but in ours 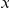 is a position. Most recently [9], 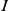 has been found to be a property of the system as well, measuring its level of ‘order’ and ‘complexity.’

## Results

### Acquiring Stable Entropy

An extreme state in a dynamical system, because it represents a maximum point in the curve, has a first derivative of 0 and is, by definition, stable to first-order perturbations, e.g. due to exterior factors such as random temperature shift. Hence a living system that is in a state of extreme Fisher information, whether a maximum or a minimum, *gains an advantage of stability*. This tends to keep it in the stable entropic state which, as we proposed, allows life to persist.

#### An extreme value of *I* stabilizes *H*

This stability property is easily shown, e.g., for the wide range of probability laws that are members of the exponential family [12] (see Supporting Information). There the entropy 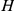 and Fisher information 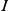 obey [13], respectively, 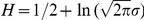 and 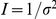. Eliminating quantity 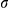 between them gives
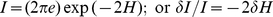
(1)after taking a differential. Since 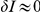 at extreme solutions then likewise 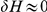. That is, *a minimum or maximum Fisher information state stabilizes entropy* 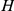 (although its internal rate of increase 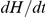 can still be finite, by (2a) below). This result clarifies the need for every stable, living system to attain an extreme level of Fisher information, whether a maximum or a minimum.

### Fisher I Limits Entropic Change

Consider a cell of mass *m* and temperature *T* shipping entropy outside its environment at a rate *dH/dt*. We have previously demonstrated [2] that there is an upper bound to the entropy change *dH*, and this depends upon the level 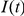 of Fisher information within the system. The relation is 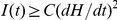, with 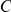 defined in Eq 2a. It is instructive to combine this with the basic entropy-energy relation 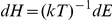, giving 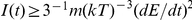 These inequalities have two ramifications depending upon whether the energy-entropy rates or the information 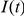 is fixed:

1. *For a fixed rate of entropy loss* 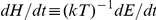 energy change by the entropy-energy relation, the minimum possible value of the information obeys
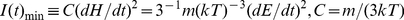
(2a)where *k* is the Boltzmann constant. These show that a cell with a low-restricted energy input rate 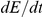, and resultingly low entropy loss rate 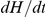, can only maintain a minimum level of information or order *I*. We propose that this state is manifested by prokaryotes, a form of life with relatively low order or complexity.
2. Or, consider *a fixed level of information* 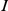. The above inequalities can be recast as
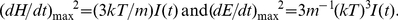
(2b)We already considered cases of minimal *I*. These described prokaryotes. Consider, now, the opposite case, that of high 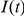, i.e. high order, complexity and function. Eqs. (2b) show that, then, even the minimum possible rates of entropy loss 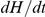 and required energy input 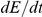 are high. The price paid for maintaining a state of high order and resultingly stable entropy is much greater required energy utilization. This state, we propose, is maintained in eukaryotes, and manifested by large genomes and the evolution of multicellularity.

Thus, the interdependence of energy and entropy provides insight into the transition from low complexity life forms to high complexity forms. It also is consistent with proposals that acquisition of mitochondria, by providing a new source of energy, was the critical factor in evolutionary transition from prokaryotes to eukaryotes. It may also provide insight into the reverse transition which is typically manifested during carcinogenesis.

### Are Information and “Order” Synonymous?

We use the words ‘order’ and ‘information’ interchangeably. As recently found [9]:

- Consider a system defined by a probability law 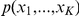. Its level of order varies linearly with its level of Fisher information *I*, i.e. order
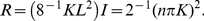
(3)Here 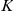 is the number of system dimensions and 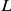 is its maximum one dimensional extension. The first equality is true in general. The second holds in the specific case of a probability law *p* that is a squared sinusoid containing 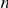 wiggles in each dimension 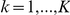. The quadratic (strong) dependence on 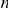 indicates that 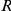 measures the level of system *complexity* as well.
- Both the order and the information are entropies, in the sense of measures of system organization that decrease with time. This thereby defines an arrow of time, perhaps quantifying the much discussed biological arrow of time.

### The structure of cells and its components optimize information and order

#### Order Required of Different Polymer States

Eq. (3) indicates that a case 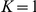 of a linear information structure (such as that encoded in the sequence of amino acids of DNA, RNA and proteins) requires intrinsically less order (and, by the preceding, less energy) to maintain its form does than a cell membrane, with 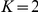. And this requires less order or energy than a composite cell, of dimension 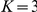. Thus, maintaining systems of high order and complexity intrinsically require cell states of both high dimensionality and, by Eq. (2b), energy resource rate.

#### Requirement of non-uniform cell structure

Unlike crystals – which have relatively uniform order throughout – living systems are heterogeneous and dynamic. Thus, even a simple prokaryote will have a highly ordered border (i.e. the cell membrane and cell wall) but a relatively disordered central structure (the fluid cytoplasm) that also contains many highly ordered large molecules such as proteins, DNA, and RNA. In other words, dynamic properties of cells seem to require spatial variations in order, e.g. in transition from the ordered cell membrane to the relatively disordered cytoplasm, to maintain a stable state far from equilibrium. The argument is as follows:

#### An information basis for Mitosis

A unique property of living systems is their ability to self-replicate and, thus, the thermodynamics of life must include this process. Indeed, mitosis and death represent key feedback mechanisms that optimize system parameters allowing system stability even while far from thermodynamic equilibrium. Consider a ‘mother’ cell developing in an environment of limited energy. A stable living system requires *dH/dt = 0*, and if *dH/dt*>0 it will dissipate and die. But, what if *dH/dt*<0? As time progresses, the complexity and order 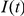 of this system will increase. However, Eqs. (2b) indicate that the higher the order *I* is the higher are the rates of entropy loss 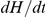 and required energy gain *dE/dt* from the surroundings. On the other hand, the surroundings can only provide a limited energy rate, call it (*dE/dt*)_0_. To what extent can order grow in the cell? The first Eq. (3) provides an answer.

As a functioning cell grows, so does its complexity *I* and, by Eq. (3), its order level *R* Then so does its required minimum energy input rate (*dE/dt*). However, this rate is inevitably limited by the cell environment to some maximum value (*dE/dt*)_0_. Let the cell order *R* grow until it reaches a level *R_0_*, where its required level (*dE/dt*)*_min_* equals (*dE/dt*)_0_. Then growth beyond level *R_0_* cannot be sustained by available energy. At this point the cell divides in half, with each ‘daughter’ containing the same set of genes as the mother. Hence each daughter has the same information level *I* as did the mother. On the other hand, each is of length *L/2*, so that by Eq. (3) each level of order is reduced, to value *R = (1/4)R_0_*. This level can once again be met by the environmental rate (*dE/dt*)_0_. Hence, now the two daughters can commence further growth in complexity; etc. Mitosis solves the problem.

#### The value of death

Unlike other stable structures in nature, living systems die. Clearly crystals and other ordered structures break down into the component parts but since they are at equilibrium, this process is slow. Living systems, however, are stably far from equilibrium so system failure will result in a rapid phase transition to equilibrium. Death, that is, represents a phase change from non-equilibrium to equilibrium.

It has been previously noted that any system far from equilibrium is inherently unstable. We propose living systems would be equally so without two critical feedback mechanisms – mitosis and death. The reward for success by proliferation and punishment for failure by death provides strong local selection for optimization of the underlying thermodynamics. Evolution thus does not emerge from biology. Rather, it is a necessary condition for the existence of living systems.

### Maximum Fisher Information

One aspect of information that is not often noted is its cost. That is, information in its storage, duplication, and utilization requires an expenditure of energy. As indicated in Eq. (2a), the local information level restricts any gain in Shannon entropy with time. *Prevention* of such a gain, i.e. maintaining a stable state of order, requires an expenditure of energy in accord with Eq. (2b). Thus, higher levels of information will require an increased expenditure of energy to maintain a stable state of order. As recently noted [10], eukaryotes maintain a state of high energy availability (compared to prokaryotes) primarily due to the development of mitochondria. These cells attain an optimum species fitness [6] by achieving a high, in fact *maximum*, *level of order and information* (also see Eq. (3)).

Thus, the energetic status of eukaryotes allows them to maintain information maximum. This is equivalent to maximizing complexity as well [9], and is manifest by a substantial increase in size and gene number in eukaryotes vs. prokaryotes. In addition, prior studies using the EPI principle (Appendix S1) have investigated the expected consequences of a system that is at an information maximum.

### Minimum Fisher Information

By contrast, prokaryotes have no specialized metabolic organelles so that energy acquisition is limited to the substrate available in the immediate environment. In such energy constrained systems, information minima are attained as the stable solution. These actually occur in contrasting scenarios of either high, or low, nutritive resource as discussed next. What mass growth laws 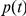 in time 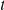 describe such system states? Note that *p(t)* is, in general, the *relative* occurrence of a given type of cell within an organ. If, for example, at time *t* a tumor occupied ¼ of an organ then the *p(t)* for cancer cells is 0.25. By the law of large numbers [13], this is also its *probability* of occurrence at a single observation. Thus, in a cancerous organ *p(t)* is the relative mass of the organ that is cancer at the time *t*, so that by the law of large numbers it also is the probability law for locating a cancer cell in the organ at a time *t*.

The solution for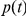 depends upon the availability of nutritive resource. For *in vitro* cases of cancer, or prokaryotes, enjoying virtually unlimited resource the minimization is unconstrained except for normalization. This gives rise [2] to temporally exponential growth laws 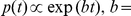
const.

### Cancer as an information transition from a maximum to a minimum

Our central hypothesis is that stability of the thermodynamic state of a living system requires the information state to be at an extremum. The transition from prokaryote to eurkaryote life forms represents a phase transition from minimum to a maximum permitted by the increased energy availability due to acquisition of mitochondria. We propose that the stepwise change from normal cells to cancer (carcinogenesis) represents a reverse phase change in which the information state transitions from a maximum to a minimum. This “information catastrophe” is manifested as the following:

#### Genomic instability

Accumulation of multiple genetic mutations is universally observed in cancers. It is estimated that typical cancer cells contain thousands of genetic changes when compared to the cells of origin. In fact, it is commonly proposed that the mutator phenotype (i.e. cells that are unable to repair DNA or chromosomal reproduction errors and, therefore, have a very high mutation rate) is required to form a cancer [14].

#### Cellular and tissue disorder

A cancer cell characteristically exhibits diminished function and disordered morphology when compared to the normal cells of origin. Similarly, tissue composed of cancer cells loses structural order (dedifferentiation and dysplasia) and normal growth constraints.

#### Inability to measure time

A hallmark [15] of cancer cells is immortality, so that their proliferation is inappropriate, within both the context of tissue formation and of their age. Telomeres are small sections of DNA at the end of each chromosome that shorten each time a normal cell undergoes mitosis. In this way the cell can “know” its age and after reaching senescence undergoes programmed death. However, cancers typically lack this measure of aging.

### Information loss and clinical cancer growth

We have previously demonstrated that the hypothesis that cancer cells asymptotically approach an information minimum allows prediction of growth dynamics. Specifically, we found that *in situ* the growth law 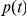 of a populations at an information minimum (i.e. either a prokaryote or a cancer cell) is a simple power law 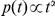, where 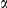 is a constant that is appropriate to the cell type [2], [7], [8]. The model predicts that, for cancer growth, α≈1.62. This prediction was compared to the growth of small breast cancers found during screening mammograms when the tumor could be observed in retrospect on two or more prior studies. Seven independent studies found that cancers exhibited power law growth with a mean value of α = 1.72 (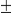
0.23) which is similar to observed in-vivo growth of bacteria with α≈2 [16]–[22].

### Why does cancer develop? – Warburg revisited

If carcinogenesis represents an information phase change from a maximum to a minimum, we must also address the system dynamics that drives such a transition. We have argued that the evolution of eukaryotes was permitted by the acquisition of improved energy dynamics that allowed a phase change to an information maximum state. We must conclude that, since a cell's energy status dictates which extreme – maximum or minimum – is favored, it is a loss of energy within a stem cell that initiates carcinogenesis. Specifically, the loss must result in energy limitation such that the cell can no longer maintain, with stability, a state of maximum information. Instead, it can only maintain with stability the state of *minimum* information that is characteristic of cancer. This does not disagree with the standard model of carcinogenesis, which assumes cancers are initiated by mutations. However, our model suggests that such mutations are a manifestation of the degradation of information that results from the energy-driven phase transition from a maximum to a minimum. Thus, while genome mutations may result in phenotypic properties that permit unrestricted growth, *our model suggests that the initiation of the mutational sequence that gives rise to carcinogenesis is the result of an acquired energy restriction*.

While this view is clearly at odds with the conventional model of carcinogenesis, it is not unprecedented. The “Warburg hypothesis” proposed that the initiating event in cancer was a loss of mitochondrial function [23]. Our prior work has [24], [25] noted that energy production in premalignant tumors can be limited by environment hypoxia (resulting from, for example chronic inflammation) and that this may be a critical step in the transition to invasive cancer. Finally, it is interesting that the role of mitochondrial dysfunction in both aging and cancer is currently a topic of considerable research interest. [26], [27].

## Discussion

The rapid development of living systems in the geological record and their continuous presence over 3.5 billion years indicates life represents a highly favorable thermodynamic state. Living systems are both remarkably stable and yet capable of developing progressively more complex (or ordered) states with time. We propose a model that explicitly includes information dynamics into the system thermodynamics can explain these properties [2]–[5], [7].

The idea of seeking a cross-disciplinary variational principle that could predict both physical and biological effects was proposed some 40 years ago by the population biologists Crow and Kimura [28] and, even before them, by Delbruck [29]. More recently, the physicist E.T. Jaynes [30] proposed a principle of maximum entropy for deriving all statistical laws of nature.

Our model views living systems as a stable, low entropy state that is, nevertheless, far from thermodynamic equilibrium. The entropy of living systems does not (unlike, for example, crystals) represent the lowest possible value of order for that system. Rather, life maintains a “goldilocks” state of entropy (not too much and not too little) that produces an optimal thermodynamic state allowing stability and self-reproduction. Death and proliferation are critical feedback mechanisms that optimize system parameters to permit stability even while not at equilibrium.

This stability requires the use of information to convert environmental energy to intracellular order. We propose the first principle of living systems is a tradeoff between (i) the internal information needed to convert environmental energy and substrate into order, and (ii) the cost of storing and using that level of order. That is, even within the ‘goldilocks” range of entropy that is compatible with life, higher levels of order will also require the system to obtain larger amounts of resource from the environment.

The stability of living systems indicates that their thermodynamic states are at extreme values, which are stable to at least first-order perturbations [31]. As to whether the extreme value is a minimum or a maximum depends largely upon the level of available energy. On this basis, the earliest living systems maintained *information minimum* because of energy restrictions. This state is still observed in prokaryotes.

By comparison, a later phase transition to information maximum occurred following acquisition of specialized energy organelles such as mitochondria. This led to a cellular phase change (viewed in the fossil record as evolution) from prokaryotes to eukaryote. The information maximum in the latter is reflected in increased numbers of genes and larger cell size and complexity. The information maximum, we propose, also allowed the emergence of multicellularity.

Conversely, we propose the evolution of a normal mammalian cell into a cancer cell represents an information phase transition from a maximum to a minimum value, probably through a number of unstable intermediates. Our model indicates this transition could be initiated by loss of energy through intra- or extra-cellular factors. In particular we note the possibility of mitochondrial dysfunction as a metabolic initiator of carcinogenesis. This is remarkably similar to the Warburg Hypothesis that was proposed over 50 years ago and is supported by more recent research [32] including studies showing mitochondrial dysfunction is closely related to aging and senescence [26], [27].

## Supporting Information

Appendix S1 Detailed explanation of Fisher Information and its properties.
(DOC)
