# Supplementary material for: Information Dynamics in Living Systems: Prokaryotes, Eukaryotes, and Cancer
Source: PLoS One. 2011 Jul 19;6(7):e22085. doi: 10.1371/journal.pone.0022085 (PMC3139603; doi:10.1371/journal.pone.0022085)
Supplement: Appendix S1 — Detailed explanation of Fisher Information and its properties. (DOC) [file pone.0022085.s001.doc]

**Appendix S1: Properties of Fisher Information**

Shift invariance

Let a required parameter $x_{0}$ be measured, as a data value$y=x_{0}+x$, with $x$ a random error obeying some probability law $p(x).$ The system is assumed to obey shift invariance, i.e. $p_{Y}(y|x_{0})=p(x)$. The data are processed arbitrarily to form an estimate of $x_{0}$. Denote the mean-squared (ms) error over many such estimates of$x_{0}$as $e^{2}$. Its *minimum* possible error $e_{\min}^{2}$ obeys [11], [12], [13]

$e_{\min}^{2}=1/I$, where $y=x_{0}+x$ , (S1)

Where $I$is the level of Fisher information about the parameter that is present in the data. Thus, a system with a high level of information gives rise to a low ms error, and vice-versa. Relation (S1) is called the Cramer-Rao inequality.

The information may be expressed alternatively as

$I\equiv\int dxp'^{2}(x)/p(x) = 4\int dxq'^{2}(x)$, $q(x)\equiv\sqrt{p(x)},$ (S2)

with $q(x)$defined as the probability *amplitude* of the system. Primes denote derivatives Information $I$has the following distinctive property that makes it well suited for predicting living systems.

Local Nature of *I*

Central to our thesis is *that life is a local phenomenon* (see Introduction). Note in this regard that either expression (S2) for$I$is a *local measure*. This agrees with the view that a cell is not a single unit but, rather, consists of two or more *local* units, each with its own value of $I$(examples are the membrane and a DNA molecule). This makes sense since the building blocks of each region are different: DNA is made up of nucleotides while the cell membrane is made up of lipids which are long carbon chains. The point is that the cell will have several internal, local probability amplitudes.

Note also that, mathematically, a “local measure” is one whose value can change drastically under local rearrangement of its points $x.$ Any such rearrangements of points$x$excite discontinuities in the curves of $p(x), q(x)$, giving local points of infinite slope$p'(x)$ or $q'(x)$, and contributing infinities to the integrals (S2) for $I$. By comparison, a *global measure*, such as the entropy

$H=-\int dxp(x)\log p(x)$ (S3)

or its corresponding summation form, does not depend upon local derivatives of $p(x)$ or $q(x)$and, hence, *does not change* its value if the points$x$are rearranged. As discussed above, life is an expression of high *local* order. On this basis, the use of a local measure such as $I$ seems optimum, and a global measure such as$H$suboptimal, for purposes of mathematically modeling its structure, i.e. complexity of order. By comparison, the use of $H$ is essential in evaluating the inputs and outputs of heat and other forms of energy from the ordered, living system. This is through the well-known equivalence $E=kHT$ equation of heat energy $E$ and entropy $H$ with $T$the temperature.

Exponential Family

The exponential family [12] of probability laws $p(x)$ will be of particular interest to us. It includes many of the most common laws, including the normal, exponential, gamma, chi-square, beta, Dirichlet, Bernoulli, binomial, multinomial, Poisson, Rayleigh and many others. Each of these has a well-defined variance value $\sigma^{2}$in$x.$ Remarkably, substitution of any of these laws $p(x)$ into (S2) gives the same result

$I=1/\sigma^{2}$. (S4)

for the Fisher information about the parameter. Intuitively, a high variance, or uncertainty, in its data defines a system that provides low order or information $I$ about the unknown parameter. This is quantified in (S4). It states that a haphazardly growing cell, with a resultingly high spread$\sigma^{2}$in values of a parameter defining its stage of growth, contains *minimum Fisher information* about that stage of growth. Or, conversely, cell growth obeying small spread$\sigma^{2}$in the parameter displays maximum Fisher information about the growth.

Through Eq. (S3), information $I$is also a measure of system order$R$which, in turn, measures system complexity [9] as well. Also, like$R$, quantity $I$is intrinsically a *system* property rather than being merely a data property. Finally, $I$ defines an arrow of time, i.e. is an entropy, monotonically decreasing after any coarse graining of the system.

Introduction to Extreme physical information

The principle of extreme physical information (main refs. [2] or [5], and uses in [3-5, 7,8] ) is an approach to deriving the probability law $p(x)$ governing a given statistical effect. It uses the principle

$I-J=$extremum, (S5)

where *I* and *J* are levels of Fisher information about a system parameter $x_{0}$ (see material above Eq. (S1)). Information $I$is that in typical data from the system, obeying (S2), and *J* is the level of information back at its source, i.e. in the system per se. Information $J$ is generally modeled based upon the user’s level of prior knowledge of the system unknown $x_{0}$ [5]. Thus, both informations $I,J$are known functionals of the unknown law$p(x)$.

In general, any observation is imperfect, i.e. it tends to contain less information than was present in the system. However, countering this effect, the extremum in (S5) is usually a minimum, so that (S5) states that although the flow $J\to I$of information that gave rise to the data is lossy, the loss is a minimum. In essence, this validates the act of taking data. With information functional forms $I,J$thereby known, the mathematical problem (S5) is generally well-defined. Depending upon whether $p(x)$is a continuous or discrete probability, (S5) may be solved respectively by either the use of Euler-Lagrange equations or ordinary differential calculus.
